# Listening in

**DOI:** 10.7554/eLife.11665

**Published:** 2015-10-21

**Authors:** Erich D Jarvis

**Affiliations:** Department of Neurobiology and the Howard Hughes Medical Institute, Duke University Medical Center, Durham, United Statesjarvis@neuro.duke.edu

**Keywords:** zebra finch (*Taeniopygia guttata*), call interactions, group communication, individual recordings, breeding stages, successful reproduction, other

## Abstract

Zebra finches communicate with each other in ways that are more complex than previously thought.

**Related research article** Gill LF, Goymann W, Ter Maat A, Gahr M. 2015. Patterns of call communication between group-housed zebra finches change during the breeding cycle. *eLife* **4**:e07770. doi: 10.7554/eLife.07770

**Image** Wireless microphone backpacks have been used to study communication between zebra finches. Photo: Susanne Seltmann

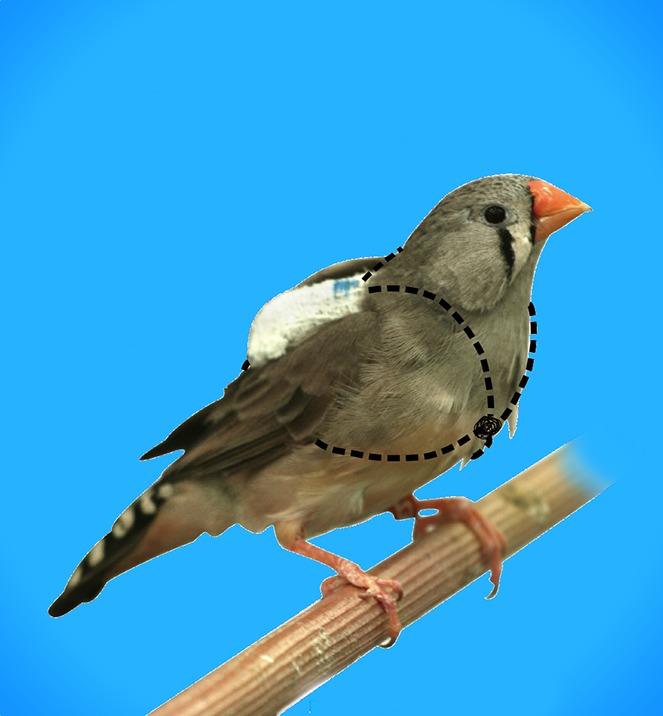

Many vertebrate species communicate with sounds. These sounds can range from simple innate calls, such as monkey alarm calls, to complex sequences of sounds that need to be learned, such as bird song and human speech. However, it has been challenging to study these more complex sounds under natural conditions because, for many years, recording technology has not been good enough to record multiple animals at once. Thus, researchers often resorted to using a combination of a single microphone and constant visual observation to study one, two or three animals at a time.

Now, in *eLife*, Lisa Gill, Wolfgang Goymann, Andries Ter Maat and Manfred Gahr of the Max Plank Institute for Ornithology report how backpacks containing miniature wireless microphones can be mounted onto the backs of zebra finches and then used to listen in as these songbirds interact with each other (Gill et al., 2015). A typical zebra finch is the size of a mouse and weighs about 15 grams, so the backpack had to be light and attached in way that did not prevent the bird from flying or copulating. The backpack, which weighed about 1 gram, was attached so that the microphone faced the animal, whereas the antenna that sent the signal to the recording apparatus pointed away from the bird (see image). The Max Planck team also developed software that automatically categorizes vocal interactions between two or more animals.

Prior to this study, the best way to record multiple animals in the same location was to use an array of microphones, followed by sophisticated sorting software to identify which animals were vocalizing, as was recently done with mice (Neunuebel et al., 2015). Last year Alexei Vyssotski and co-workers in Moscow and Zurich used microphones attached to zebra finches for the first time (Anisimov et al., 2014): however, the data were stored on a chip in the backpack, and had to be manually uploaded to a computer later, rather than being transmitted to the computer wirelessly in real time as done by Gill et al.

The subsequent experiment designed by Gill et al. is reminiscent of a reality television show. Imagine having a group of single young men living in a house for an extended period time, and a group of single young women living in a different house. Then select four of the men and four of the women, attach wireless microphones to them, move them to a large house in the tropics with four bedrooms, and record every word they say for 20 days. This is what was done with the birds, except that the house was a large, hot and humid aviary, and the rooms were nest boxes, provided with nest material nearby. No rules were enforced on the animals, so they were free to wander, socialize, mate and fight as they wished.

Gill et al. found that the communications between the birds changed over the course of the 20 days, partly depending on who paired with whom. At first all the birds produced a flurry of long distance calls, and each bird responded a lot to all other birds. After several days, as soon as they found the nest material, individual birds started to form opposite-sex pairs, communicating with each other much more (by a factor of five or six) than with the other birds. The birds in a pair also tended to use ‘cackle and whine’ calls to communicate with each other, but not with other birds. ‘Tet’ calls were produced more often by males during unpaired and non-nesting social contexts, whereas ‘stack’ calls were produced more often by females during unpaired and nest defense contexts.

A remarkable finding was that after 15–20 days, the male–female pairs that used the same call types to communicate with each other were more successful in laying and incubating eggs. Those that communicated more often with different call types were unsuccessful mates. These findings indicate that proper communication is important for forming successful pair bonds and producing offspring, and show that there is more to the chatter of birds than randomly produced calls.

Relatively few animals are capable of vocal learning: those that are include songbirds, parrots, hummingbirds, humans, bats, elephants, dolphins and seals (Petkov and Jarvis, 2012). In zebra finches, as in many other songbirds that live in temperate climates, males have the ability to learn how to produce novel vocalizations, whereas females have lost the this ability (Odom et al., 2014) for both song and calls (Simpson and Vicario, 1990). However, the results of Gill et al. could mean that although female zebra finches cannot imitate new sounds, they still learn when and where to produce their innate sounds through social learning.

The results of the Max Planck team might stimulate further research on other species that vocalize, including non-human primates such as marmosets, macaques and chimpanzees: both the males and females of these species can only produce innate vocalizations but, like female zebra finches, they are capable of learning when and where to produce these sounds through social experience (Petkov and Jarvis, 2012; Takahashi et al., 2015). In the long term this combination of wireless technology and sophisticated software may help us learn more about the rules that govern vocal communication in animals.
